# Perspectives of patients, carers and mental health staff on early warning signs of relapse in psychosis: a qualitative investigation

**DOI:** 10.1192/bjo.2019.88

**Published:** 2019-12-12

**Authors:** Stephanie Allan, Simon Bradstreet, Hamish J. McLeod, John Gleeson, John Farhall, Maria Lambrou, Andrea Clark, Andrew I. Gumley

**Affiliations:** Student, Glasgow Institute of Health and Wellbeing, University of Glasgow, UK; Trial Manager, Glasgow Institute of Health and Wellbeing, University of Glasgow, UK; Professor, Glasgow Institute of Health and Wellbeing, University of Glasgow, UK; Professor, Australian Catholic University, Australia; Associate Professor, La Trobe University, Australia; Research Assistant, Australian Catholic University, Australia; Research Assistant, Glasgow Institute of Health and Wellbeing, University of Glasgow, UK; Professor, Glasgow Institute of Health and Wellbeing, University of Glasgow, UK; see Acknowledgements.

**Keywords:** Implementation, schizophrenia, clinical decision-making, qualitative research

## Abstract

**Background:**

Relapse prevention strategies based on monitoring of early warning signs (EWS) are advocated for the management of psychosis. However, there has been a lack of research exploring how staff, carers and patients make sense of the utility of EWS, or how these are implemented in context.

**Aims:**

To develop a multiperspective theory of how EWS are understood and used, which is grounded in the experiences of mental health staff, carers and patients.

**Method:**

Twenty-five focus groups were held across Glasgow and Melbourne (EMPOWER Trial, ISRCTN: 99559262). Participants comprised 88 mental health staff, 21 patients and 40 carers from UK and Australia (total *n* = 149). Data were analysed using constructivist grounded theory.

**Results:**

All participants appeared to recognise EWS and acknowledged the importance of responding to EWS to support relapse prevention. However, recognition of and acting on EWS were constructed in a context of uncertainty, which appeared linked to risk appraisals that were dependent on distinct stakeholder roles and experiences. Within current relapse management, a process of weighted decision-making (where one factor was seen as more important than others) described how stakeholders weighed up the risks and consequences of relapse alongside the risks and consequences of intervention and help-seeking.

**Conclusions:**

Mental health staff, carers and patients speak about using EWS within a weighted decision-making process, which is acted out in the context of relationships that exist in current relapse management, rather than an objective response to specific signs and symptoms.

Relapse influences the long-term course of psychosis with rates following a first episode accumulating to 54% at 3 years^1^ and 80% at 5 years.^2^ The economic costs of treating relapse are significant.^3^ Furthermore, relapse increases psychological distress and demoralisation in patients^4^ and disrupts important interpersonal relationships such as those with carers.^5^ Lack of acceptance of treatment and unplanned discontinuation of antipsychotics are predictors of relapse^1^ reflecting poorer engagement with mental health staff.^6^

One way of addressing risk of relapse is monitoring early warning signs (EWS). EWS monitoring is well established for the detection of relapse, but evidence for routine implementation is poor.^7^ This may in part be because of the relatively poor sensitivity of formal EWS monitoring (median sensitivity 61%)^8^ and fear of relapse^9^ leading to avoidance of help-seeking.^4^ Lack of meaningful relationships between patients and mental health staff prevents implementation of crisis care plans and is a missed opportunity for shared decision-making.^10^ Therefore, successful implementation of early signs monitoring to detect and prevent relapse not only rests on being able to accurately predict relapse, but also on the quality of interpersonal interactions, communication and shared decision-making, including with families and carers.^11^

## Study aims

The EMPOWER trial was simultaneously conducted in both the UK and Australia. The primary aim of this study was to develop a multiperspective theory of how EWS are utilised by patients, staff and carers to inform the future implementation of a clinical trial of digital technology for relapse detection and prevention (EMPOWER Trial, ISRCTN: 99559262). The selection of research questions followed the Medical Research Council^12^ framework for developing and evaluating complex interventions.

## Method

### Design

A qualitative focus group design was chosen to gain insight into participants' perspectives, experiences and expectations.^13^ Using focus groups enabled respondents to interact with and respond to the ideas and comments of other participants.^14^ Following best practice guidelines,^12^ we used a theoretical framework to guide our focus group schedule. An interview schedule was informed by normalisation process theory (an implementation theory that helps model the attitudes, behaviours and reflections that affect the integration of new complex interventions into routine care)^15^ was developed to explore stakeholders' expectations. We planned to use normalisation process theory to explore how mental health staff, carers and patients made sense of EWS, how they engaged with them, the actions they took in relation to EWS and how effective they thought EWS were in managing relapse.

### Sampling and recruitment

Staff who support people with psychosis were recruited from community mental health services (CMHS) in Glasgow, UK and Melbourne, Australia. Staff were invited to take part through the research team making contact with clinical team leaders in all eligible CMHS within both health boards. Patients were recruited to take part in focus groups through a direct approach by mental health staff and posters placed in support organisations for people affected by mental health problems. Patient-participants were eligible if they were in contact with a local CMHS; had experienced a relapse in the past 2 years; had a diagnosis of a psychosis spectrum condition and were able to provide informed consent.

Self-identified carers for people with psychosis were recruited by the research team advertising through posters and word of mouth in mental health services and support groups. Participants included 88 mental health staff, either working in the National Health Service in the UK (*n* = 54, nine focus groups) and NorthWestern Mental Health service in Australia (*n* = 34, five focus groups). Twenty-one patients were recruited from local mental health services in the UK (*n* = 5, three focus groups) and Australia (*n* = 16, four focus groups) and 40 carers from UK (*n* = 20, two focus groups) and Australia (*n* = 20, three focus groups). To maximise participant anonymity, we did not collect any demographic data beyond whether the participant was a carer, patient or mental health clinician.

### Procedure

The authors assert that all procedures contributing to this work comply with the ethical standards of the relevant national and institutional committees on human experimentation and with the Helsinki Declaration of 1975, as revised in 2008. All procedures involving patients were approved by the West of Scotland (REC/16/WS/0042) and Melbourne Health (REC/15/MH/344) Ethics Committees. Written informed consent was obtained from all participants. Carers and patients received £20/$40 and participated in private rooms within mental health support organisations. Staff received no financial reimbursement and participated in their place of work during working hours. Following a short presentation about EMPOWER that covered trial rationale, design and key aspects of the intervention, 25 focus groups were conducted locally following the topic guide. Focus groups were conducted by the authors A.I.G., H.J.M., J.F. and J.G. (clinical psychologists), A.C., M.L. and S.A. (research assistants) and S.B. (trial manager), who are all trained in qualitative methods, between 20 July 2016 to 6 September 2017. Both UK and Australian teams met remotely around once a week via teleconference to discuss the study. Focus group length ranged from 57 min to 2 h 9 min. The focus groups were audio recorded and then transcribed verbatim with transcripts stored on a secure server. NVIVO software was used^16^ to perform analysis.

### Analysis

Constructivist grounded theory assumes that the phenomenon in question is interpersonally constructed and context dependent.^17^ The analysis followed the constructivist grounded theory approach as outlined by Charmaz.^17^ Our philosophical stance was influenced by structural symbolic interactionism, which posits that context is closely linked to how social or organisational roles of each stakeholder influences their daily life.^18^ For example, being a mental health professional comes with a set of normative expectations regarding the nature of their mental health expertise, expected actions and role-driven behaviours.^10^ This was considered important as different stakeholders may have different perspectives on psychosis management.^19^ Constructivist grounded theory posits^17^ that themes do not emerge from the data but are constructed as part of a reflexive analytic process. Therefore, themes will be reported as such.

Focus group transcripts were coded line-by-line by primary coder S.A. (a PhD student researching digital interventions for psychosis) through an inductive process, developing open codes that summarised the transcripts. Categories were then formed by repeatedly comparing open codes to see if these could be linked together. During the final theoretical coding stage, data from all three groups were repeatedly compared to see if categories could be linked together as a higher order unifying or overarching theme, or if there were differences between groups.

There is no prescriptive method for ensuring quality in qualitative analysis,^20^ therefore, reporting of results followed good practice guidelines.^21^ For example, we made our philosophical position (constructivism) clear for the reader and our intentions behind the study (exploring existing context in advance of implementing an intervention) transparent. Furthermore, S.A. kept reflexive memos throughout the study, which recorded how the influence of researcher characteristics and experiences may have shaped analysis. Analysis was triangulated by discussion with academic clinicians (A.I.G. and H.J.M.). Only data relevant to the aims of study (understanding how EWS management is currently sustained in context by three stake-holding groups) were included in this paper. Member checking (where participants check over themes proposed by the researcher as an interpretation validity check)^22^ was not undertaken. The quotes chosen are illustrative of the most common constructions within the data. See Appendix 1 for an overview of the overarching themes and subthemes.

## Results

### Weighted decision-making

We constructed an overarching theme termed weighted decision-making, reflecting a process acted out by different stakeholders' responses to the uncertain context of EWS. Throughout all groups, weighted decision-making was constructed as a process that emerged from interactions between patients, staff and carers (if a person had one). Weighted decision-making was strongly linked to risk appraisal, with each group having their own distinctive (and sometimes shared) experiences of the harms and benefits of acting on EWS as a strategy to prevent relapse. This overarching theory comprised four key themes: the apparent consensus around EWS; meaning and consequences of EWS identification; experience as expertise; and EWS decision-making processes, each of which is now described and explained in turn.

### The apparent consensus around EWS

There was consensus across stakeholders that EWS were experiences or behaviours that are taken to indicate risk of relapse, and that identifying EWS was a potentially useful way to understand changes in well-being and to allow for early intervention. Patients described a variety of personal experiences labelled as EWS. Their descriptions suggested that EWS function as a barometer for recognising changes in well-being.

Researcher 2: ‘And how helpful would you say it is to kind of monitor early warning signs?’

Participant 2: ‘It's important. It's important for your wellbeing. See how you feel the next morning. See how your health is, your mindset. It's very important.’ (Patient group 6, Australia)

Carers reflected on the function of EWS as a means to identify and act in order to avert mental health crises. Carers valued monitoring early signs as a basis for preventing a potential relapse.

‘You can stop it from escalating and into a full-blown episode. You can see when it's coming on. They can increase the medication or encourage them to see the doctor or something like that. There's lots of things you can do. But, you know, once they get too sick, then it gets more difficult, they get more suspicious.’ (Participant 6, Carer group 4, Australia)

Mental health staff reported that EWS offered an indicator that things were starting to break down for a patient and signalled when staff believed that intervention was needed. Staff appeared to believe that if EWS were noticed and acted upon, then there was an opportunity that relapses could be managed.

‘I think it's their behaviours; you know, if there's a sudden change or you know, that if you've done your relapse prevention and they have identified relapse triggers, then the person's starting to do them.’ (Participant 1, Staff centre 7, UK)

### Meaning and consequences of EWS identification

Although there was broad agreement by stakeholders on the function of EWS as a tool to detect and ameliorate risk of relapse, each group articulated distinctive expectations regarding the possible sequence of events following the recognition of EWS. These distinctive expectations were closely linked to the meanings of relapse for each stakeholder group.

Carers generally valued EWS, however, our analysis suggested that the benefits of identifying EWS came with the possible consequence that a loved one could feel harmed from subsequent interventions. Many carers described traumatic experiences connected to relapse (such as an admission to hospital) and their reluctance to involve services. Although carers recognised the value of recognising and acting on EWS, they also feared the potential impact of acting on these in terms of the impact on their relationship with their loved one or the impact of experiences such as being put in hospital upon their loved ones.

‘I don't want to do anything that's going to push her, you know because there's already part of that whole sectioning process, and that is a huge thing to go through that in a family dynamic.’ (Participant 5, Carer group 2, UK)

Many patients spoke in great detail about the personal distress caused by experiences of psychosis in the context of a relapse. Such experiences were commonly described by patients, which suggested that relapse represented a threat to personal well-being. Patients often feared that relapse would also be associated with a series of consequences including the expectation that letting others know about EWS could result in a potential threat to their autonomy such as being made to go to hospital.

‘Sometimes you don't want to say anything in case you get a negative response. I don't want to go back to the hospital.’ (Participant 2, Patient group 2, UK)

A sense of relapse being somewhat inevitable was observed across the staff focus groups.

Staff recognised that identification of EWS and relapse prevention was an expected part of their role as clinicians. Therefore, the importance of EWS monitoring as a basis for minimising the impact of relapse was generally seen as an essential part of staff responsibilities. However, staff were open to the possibility that foregrounding relapse prevention in such a risk averse manner might not accord with how patients want to manage their own well-being.

‘You are not going to prevent relapse – relapse is always going to be there. On some sort of scale, even people who are well have momentary relapses.’ (Participant 1, Staff centre 1, UK)

‘I reckon like this is a massive generalisation but I think we [staff] are probably more conservative in the sense that we would probably do more to prevent a relapse but sometimes maybe you can see the consumers’ situation where they will entertain a few more risks, you know – around the potential for relapse if they think that some other benefit like they get to do something else in their life – whereas we are probably a bit more conservative.’ (Participant 5, Staff centre 12, Australia)

### Experience as expertise

The nature of risk appraisals linked to EWS expressed by different stakeholders appeared distinctive and reflected contrasting concerns and different types of knowledge and experience. Patients described a dual process of making sense of their own experiences and of how others (particularly mental health staff) would interpret their experiences. This was heavily influenced by their own risk assessment: acknowledging the personal threat a relapse posed and then assessing the external threat of how other people would respond. Patients embedded their appraisals in their previous experiences when appraising EWS disclosure risk. In addition, patients perceived that their experiential expertise in appraising the risk of EWS could be downplayed by staff.

‘We hate hospital, but we also want to be as honest as we can and often we want to be able to manage our own symptoms too. We don't want to be medicated up to our eyeballs.’ (Participant 1, Patient group 5, Australia)

For carers, their expertise in assessing the EWS risk in this context came from knowledge gained through their close contacts. Carers often described themselves as being able to successfully contextualise the risk of EWS through knowing their loved one.

‘my sister's now, what, fifty-two, so she was diagnosed when she was twenty-one. And why I say I'm her carer, is I recognise the signs.’ (Participant 3, Carer group 4, Australia)

Carers also reported frustration that staff sometimes did not value carer assessment of the risk posed by EWS. Many carers believed that staff did not recognise the value of their knowledge, experience and expertise.

‘Well I don't like the fact that I get told “your son is doing really really well, really really really” and I phone up and say “I'm really concerned”.’ (Participant 2, Carer group 2, UK)

Staff reflected on their expertise in recognising and acting upon EWS. This appeared formalised in professional mental health guidelines and policies reflecting both their broader clinical expertise and also their more intimate knowledge of a patient. They reflected on the uncertainty and complexity of acting in response to early signs and the importance of being able to personalise their response to EWS.

Participant 2: ‘we're kind of conscious that we'll be trying to fit it to the individual. Obviously, people's experiences with something might work better for some people. But I'd imagine it'd be pretty similar with a few provisions maybe.’

Participant 3: ‘There an Integrated Care Pathway and kind of all those guidelines.’ (Staff centre 7, UK)

‘you can only do what you can do as a key clinician in terms of you know those policy procedures we've just discussed and there's a whole other gamut of influences that might impact on a consumer that are out of our scope to influence I guess. So, um, I think that's … it's tricky and every consumer is completely different and has different warning signs based on potentially their diagnosis or potential harm so it's yeah. I guess when you are actually addressing those EWS – it really just depends.’ (Participant 1, Staff centre 12, Australia)

Staff valued their individualised knowledge built up through their relationships with patients. In the absence of this, staff then placed their expertise in a team context, utilising colleagues' insights and case notes for developing and evidencing their risk assessment of EWS. The actions reported by staff implied that broader staff knowledge was regarded as a reliable and trustworthy source of information.

‘I'm just thinking, what would I do in a crisis phone call? So, I would look at notes, try and speak to the person. I would go to that team and say, “who knows this person?” So, I would be trying the collective formal and informal consciousness of the team. Because I don't know all the answers.’ (Participant 1, Staff centre 3, UK)

If staff recognised behaviours they believed were EWS (without a patient reporting EWS experiences to staff) then this appeared to be taken as a ‘lack of insight’ and an indicator of increased risk of a relapse occurring. This commonly resulted in staff believing they needed to take ownership of clinical risk assessment, which appeared to impede sharing decision-making.

‘There's Staying Well plans and stuff like that. People, we get to sit down and talk about that side, and what happens when they become unwell and things like that. But I've never really had a patient phone me saying “listen, I've referred back to this plan, and I'm starting to get some of these symptoms”. You know. It's usually way past that and you pick it up yourself because they just don't have the insight to notice.’ (Participant 4, Staff centre 9, UK)

### EWS decision-making processes

For staff, intervention decisions were based upon a risk gradient. For example, if EWS were perceived to be low risk (in this context – a threat to patient well-being but which could not be ruled out as a false positive), then staff interventions focused on helping the patient manage their experiences. If the risk was perceived to be greater (in this context risk being severe detriment to patient well-being and more indicative of a relapse event), then the staff role shifted from relapse prevention to relapse management where intervention options included enforced treatment.

‘there will be steps to take to ensure the consumer isn't getting to the point where they are really unwell – being able to prevent that pretty much – most of it, all of it, getting in contact, you know. Getting input from the medical team – even using legal measures such as a temporary community treatment order or a system those things …, yeah. It's different for everybody, but there are ways where we can really try and manage someone.’ (Participant 3, Staff centre 12, Australia)

Decisions were contextualised by constraints on mental health service provision particularly responsibilities for large case-loads. Staff reported having to invest more time with patients who they perceived to have the greatest likelihood of experiencing relapse and feeling dissatisfied with routine practice. This was felt to have an impact on the quality of working relationships and the ability to respond proactively to EWS.

‘What we find difficult as nurses is massive caseloads and trying to maintain quality of care trying to make sure things like that are all up to date so it's hard it's nice in theory to say “oh this is what happens and this always happens” but we'd love it to always happen but sometimes we don't have time to do that and it's there chasing you every day and you're thinking oh my god. Best practice. Core standard. Every patient would have that, but reality is we don't often get the time to do it for everybody.’ (Participant 1, Staff centre 1, UK)

Decision-making for patients was constructed as a process of weighing up the personal benefits and risks of disclosure of EWS. The expectation that other people would overreact was frequently described as a barrier to help-seeking. Patients described how their decision-making processes were shaped by these sources of uncertainty associated with disclosure.

‘I get a bit scared to tell people about the early signs. Because you don't want people to blow it out of proportion and then they're staring at you and watching your every move. I don't like that; I like my privacy. And so I like…I don't know.’ (Participant 1, Patient group 1, UK)

Some patients spoke about times when staff responses did not result in an expected level of support to help them manage distress. This meant that patients were left to manage distress alone, left them feeling dejected and less incentivised towards future help-seeking.

‘it's really hard to get to help when you're in psychosis, people don't want to take on the responsibility of helping you and it's not always very easy to ring triage and then get the appropriate response that you want.’ (Participant 3, Patient group 4, Australia)

The struggles expressed by patients with respect to the personal risks of help-seeking were also reflected by carers who did not believe staff were likely to have access to all the relevant information needed for optimal decision-making. Carers reported that they could provide context and detail especially in response to non-disclosure by patients.

Participant 2: ‘We often have the feeling that we want to ring them up and say “but all this is going on, and you probably don't know about it” or “she's probably not telling you the history of what's happened beforehand”.’

Participant 4: ‘Yeah. That's why it's really important to say “This is what's really going on”.’ (Carer group 4, Australia)

## Discussion

This study aimed to create a multiperspective theoretical account of how EWS are experienced and acted out in routine practice. Relapse into psychosis has major ramifications for everyone involved. However, to the best of our knowledge, the context in which EWS are identified and acted on has not previously been described in detail from the perspectives of mental health staff, patients and carers. Self-management interventions for severe mental illness have been reported to generally improve mental health outcomes but the evidence is mixed for relapse prevention.^23^ However, this was noted to be in contrast to a previous meta-analyses that focused on schizophrenia^24^ where self-management interventions (including EWS monitoring) were associated with a significant reduction in relapse events. A call has been made for research that is focused on how to understand and overcome barriers to the implementation of self-management interventions.^23^ This study represents the first large-scale qualitative investigation of how EWS are understood and experienced by staff, patients and carers across two geographically distinct healthcare systems.

### Weighted decision-making

Relapse was described as a negative event throughout the focus groups with no stakeholders in these specific focus groups describing relapse as potentially positive, which may imply relapse is perceived as a persistent and ongoing threat. All groups seemed to recognise EWS and emphasised their possible value for relapse prevention. However, recognition of and acting on EWS were constructed in a context of uncertainty – in line with previous research from early intervention populations.^25^ Within current relapse management, a weighted decision-making process described how stakeholders weighed up role-congruent^26^ consequences of relapse alongside role-congruent risks and consequences of intervention and help-seeking. A key finding from our qualitative analysis was that responses to EWS appeared linked to risk appraisal and there are differences in how risk is appraised by staff, carers and patients that are closely related to participants' previous experiences and for staff, their professional role.

Similar to findings from previous research,^27^ carers seemed concerned about relapse. Carers weighed the risk posed by EWS of a potential relapse within the context of their close personal knowledge about the person they care for. Carers described wanting to provide supplementary or countervailing information to predict relapse and improve clinical decision-making. However, they reported that their expertise and EWS assessment were often dismissed and devalued by staff. These findings resonate with previous research showing that carers believe they have excellent knowledge of the EWS of relapse from close relationships, but do not feel staff value their knowledge as clinically relevant.^28^ Furthermore, carers disclosing EWS to staff may come with a risk of undermining relationships with patients.

The uncertainty about the degree to which staff may respond was a key factor, which weighed into patients' decisions about help-seeking. This was in contrast to staff expectations that failure to help-seek arose from a lack of insight. For some patients, the potential losses arising from seeking help (such as loss of autonomy) outweighed the potential gains in terms of preventing a deterioration in well-being – echoing previous work by Sibitz *et al*.^29^ However, some patients also spoke about not receiving adequate support from mental health services when they were struggling, despite promptly reporting EWS.

Broader contextual factors influenced decision-making. Staff spoke about EWS as an important opportunity to prevent or minimise relapse, but felt that in reality they were constrained by high case-loads and inadequate resources – leading to an emphasis on crisis interventions at the cost of developing close working relationships, critical to anticipating and supporting relapse prevention. Lower staff continuity has been linked to worse clinical outcomes in schizophrenia,^30^ and inadequate staffing appears linked to increased use of restrictive practices over de-escalation techniques, because these are considered more time efficient in poorly staffed wards.^31^ Research on clinical decision-making is stated to be difficult to conduct because of the dynamic environment of applied settings.^32^ This analysis suggests EWS are utilised within a particularly dynamic and intersubjective decision-making process, with stakeholders valuing different outcomes. For example, patients may value personal autonomy over the clinical stability typically valued by staff. Although these findings are from qualitative data and should be interpreted with caution, they may offer a theoretical basis for exploring applied clinical decision-making further. For example, discrete choice experiments^33^ would allow the relative value of costs and benefits for different stakeholders to be empirically tested.

### Limitations

With qualitative research it is not possible to make comment on the generalisability of any findings. Furthermore, all participants volunteered to take part within focus groups, which raises the possibility that we may have missed important perspectives from those who did not participate. Although focus groups allowed for participants to interact together and discuss topics, it may be the case that some participants could feel uncomfortable contributing contradictory viewpoints. Individual interviews may have been more appropriate for discussing potentially sensitive topics such as EWS.

The real-world clinical context of EWS usage comes from interactions between mental health staff, carers and patients. However, we spoke to these groups in isolation from each other, which may have had an impact on the results. Also, although at a conceptual level we observed no differences in how people understood and use EWS between Australia and UK, we did not explicitly explore the way in which the distinctive health systems that contextualise practice might influence stakeholders' views and experiences.

Additionally, staff, carer and patient participation were unbalanced, with lower numbers of patients participating. Our approach to recruiting mental health staff enabled us to systematically approach all local CMHS in our two study sites. However, we were unable to have such a systematic approach to user and carer participation and our lower rates of participation reflect this. Furthermore, it should be acknowledged that many patients live alone in the community without support from a carer.

Not collecting demographic data was important to better protect participant anonymity and this was a pragmatic methodological choice, however, we acknowledge it is a limitation for readers in interpreting the data.

### Implications for mental health practice

Qualitative methods have been found to be helpful in uncovering ‘ruptures in communication’, wherein differences in how doctors and patients understand medical problems can lead to distress and dissatisfaction with care.^13^ In highlighting how mental health staff, patients and carers described their experiences, it seems EWS are used within a weighted decision-making process, which is acted out in the context of relationships, rather than an objective response to specific signs and symptoms. However, there were marked differences in how groups spoke about this decision-making process. Patients and carers reported that staff sometimes did not appear to value their knowledge about EWS and self-management. This may be part of a broader issue, where the subjective experiences of patients and carers are perceived to be less clinically useful because these are perceived to be at risk of bias.^34^ These findings echo previous qualitative research examining implementation of joint crisis care plans, which found that staff prioritised risk assessment in accordance with their professional roles at the cost of addressing patients' priorities for their treatment and care.^35^

EWS-based intervention development and implementation may be enhanced by better utilising the knowledge of carers and patients who bring expertise to shared decision-making. For example, interventions that gather data from patients and/or carers may reduce clinical uncertainty by placing EWS in a meaningful context. The sharing of data showing temporal well-being changes within this weighted decision-making process could influence interactions with key clinicians and reduce uncertainty about the potential negative impact of help-seeking (for all groups) by allowing for predictable alerting of EWS.

Relationships appear to be the context in which weighted decision-making involving EWS is both acted out and understood. These results suggest that uncertainty of how others will behave may create a barrier to early intervention. EWS-based interventions may be better implemented if they address this uncertainty by providing a clearer stepped care pathway, for example through a focus on shared decision-making.^36^ In order for EWS-based interventions to be a ‘good fit’ within the context of current relapse management, interventions should aim to change the interpersonal interrelationship behaviours of staff, patients and carers (where applicable), rather than targeting a single social actor.

## Data Availability

S.A., A.I.G. and H.J.M. have ongoing access to the transcripts.

## Appendix 1

### Themes

**Table:**

Dominant theme	Subthemes
1. 1. Weighted decision-making	1.1 The apparent consensus around early warning signs
	1.2 Early warning signs decision-making processes
	1.3 Experience as expertise
	1.4 Meaning and consequences of early warning signs identification
